# Characteristics of free air carbon dioxide enrichment of a northern temperate mature forest

**DOI:** 10.1111/gcb.14786

**Published:** 2019-09-11

**Authors:** Kris M. Hart, Giulio Curioni, Phillip Blaen, Nicholas J. Harper, Peter Miles, Keith F. Lewin, John Nagy, Edward J. Bannister, Xiaoming M. Cai, Rick M. Thomas, Stefan Krause, Michael Tausz, A. Robert MacKenzie

**Affiliations:** ^1^ Birmingham Institute of Forest Research (BIFoR) University of Birmingham Birmingham UK; ^2^ School of Geography, Earth and Environmental Sciences University of Birmingham Birmingham UK; ^3^ Yorkshire Water Bradford UK; ^4^ Brookhaven National Laboratory Upton NY USA; ^5^ Department of Agriculture, Science and the Environment School of Medical, Health and Applied Sciences Central Queensland University Rockhampton Qld Australia

**Keywords:** deciduous, elevated carbon dioxide, FACE, oak, performance, *Quercus robur*, United Kingdom, Woodland

## Abstract

In 2017, the Birmingham Institute of Forest Research (BIFoR) began to conduct Free Air Carbon Dioxide Enrichment (FACE) within a mature broadleaf deciduous forest situated in the United Kingdom. BIFoR FACE employs large‐scale infrastructure, in the form of lattice towers, forming ‘arrays’ which encircle a forest plot of ~30 m diameter. BIFoR FACE consists of three treatment arrays to elevate local CO_2_ concentrations (e[CO_2_]) by +150 µmol/mol. In practice, acceptable operational enrichment (ambient [CO_2_] + e[CO_2_]) is ±20% of the set point 1‐min average target. There are a further three arrays that replicate the infrastructure and deliver ambient air as paired controls for the treatment arrays. For the first growing season with e[CO_2_] (April to November 2017), [CO_2_] measurements in treatment and control arrays show that the target concentration was successfully delivered, that is: +147 ± 21 µmol/mol (mean ± *SD*) or 98 ± 14% of set point enrichment target. e[CO_2_] treatment was accomplished for 97.7% of the scheduled operation time, with the remaining time lost due to engineering faults (0.6% of the time), CO_2_ supply issues (0.6%) or adverse weather conditions (1.1%). CO_2_ demand in the facility was driven predominantly by wind speed and the formation of the deciduous canopy. Deviations greater than 10% from the ambient baseline CO_2_ occurred <1% of the time in control arrays. Incidences of cross‐contamination >80 µmol/mol (i.e. >53% of the treatment increment) into control arrays accounted for <0.1% of the enrichment period. The median [CO_2_] values in reconstructed three‐dimensional [CO_2_] fields show enrichment somewhat lower than the target but still well above ambient. The data presented here provide confidence in the facility setup and can be used to guide future next‐generation forest FACE facilities built into tall and complex forest stands.

## Graphical abstract

The Birmingham Institute of Forest Research (BIFoR) has built and operated a free‐air carbon dioxide enrichment (FACE) facility in deciduous forest in the United Kingdom. BIFoR FACE aims to elevate the local atmospheric carbon dioxide concentrations of a temperate, mature, oak woodland, to mimic those predicted for 30–50 years into the future. The facility target of 150 parts per million (ppm) above ambient (currently ~405 ppm) was reached in 97% of the operation time in the first year of operation (2017). With performance of the facility characterized, researchers can now investigate how this ecosystem responds to elevated CO_2_.

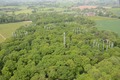

## 1 INTRODUCTION

The ‘greening’ of the terrestrial surface across planet Earth has been driven by changes to the dynamics of vegetation and their interactions, to a large extent, with increasing levels of carbon dioxide (CO_2_) in the atmosphere (Forzieri, Alkama, Miralles, & Cescatti, 2017; Zhu et al., 2016). The land carbon sink currently absorbs 20%–30% of CO_2_ released by human activities (Le Quéré et al., 2018) and a large proportion of this absorption is by woody vegetation (Gaubert et al., 2019). This sink activity is largely ascribed to the fertilization effect of increasing atmospheric CO_2_ concentrations (Schimel, Stephens, & Fisher, 2015), especially through the stimulation of growth and carbon sequestration in established, mature forest ecosystems (Luyssaert et al., 2008). However, the future magnitude of the land carbon sink, as atmospheric CO_2_ continues to increase (at least until mid‐21st century), is uncertain. Modelling of future C‐uptake rates ranges from 0% to 30% of human CO_2_ emissions, across the suite of Earth systems models used by the Intergovernmental Panel on Climate Change, Working Group 3 (Friedlingstein et al., 2014).

The uncertainty in the sensitivity of the land C sink to increasing atmospheric CO_2_ is due, in large part, to a lack of experimental data on mature forest ecosystems under future elevated [CO_2_] (e[CO_2_], Ellsworth et al., 2017; Norby et al., 2016). In the northern hemisphere, where about 40% of the net uptake occurs, highly seasonal mature forests dominate the land carbon sink (Luyssaert et al., 2008). Our experimental knowledge of how such forest ecosystems respond to further increases in [CO_2_] is based on few ‘first‐generation’ Free Air CO_2_ Enrichment experiments (FACE), either on young, vigorously growing forest plantations (Hendrey, Ellsworth, Lewin, & Nagy, 1999; Norby et al., 2006) or on small seedlings or saplings (e.g. Dickson et al., 2000; Kubiske et al., 2015; Smith et al., 2013). A somewhat different (‘WebFACE’) free‐air methodology targeted canopy exposure of mature trees, but did not quantify the CO_2_ field around the treated trees (Klein et al., 2016) and was not suitable for biogeochemical budget studies.

Since the closure of important ‘first‐generation’ forest FACE experiments—‘Duke FACE’ in an evergreen loblolly pine stand (Hendrey et al., 1999), the Oak Ridge National Laboratory FACE in a young deciduous sweetgum plantation (Norby et al., 2006), and the AspenFACE that followed aspen and poplar seedlings over a decade (Dickson et al., 2000; Kubiske et al., 2015)—the scientific community has advocated for large‐scale, ecosystem‐plot‐sized FACE experiments in important forest ecosystems (Calfapietra et al., 2010; Norby et al., 2016).

The ‘EucFACE’ experiment in an open, Mediterranean‐type sclerophyll forest in Australia (Drake et al., 2016) is the first such ‘second‐generation’ forest FACE, which has been operating since September 2012; the Birmingham Institute of Forest Research (BIFoR FACE), which is the focus of this study, is the second (Norby et al., 2016). The forest stand in BIFoR FACE has the most complex canopy structure of all forest FACE experiments to date, dominated by up to 25 m tall mature pedunculate oak (*Quercus robur*), with distinct mid‐ and understoreys formed mainly by sycamore maple (*Acer pseudoplatanus*) and common hazel (*Corylus avellana*), as well as dense ground cover vegetation (Norby et al., 2016). As a deciduous forest ecosystem, it has very variable leaf area index (LAI) during the active vegetation and CO_2_ fumigation period, from very low at the spring flush to very high LAI (>5 m^2^/m^2^; Norby et al., 2016) during the main summer assimilation period (MacKenzie et al., 2019).

The structural and temporal characteristics of the BIFoR FACE forest pose specific problems for the CO_2_ exposure system, which are not directly comparable to those in ‘EucFACE’, with its evergreen and much sparser canopy (Duursma et al., 2016). Oak Ridge National Laboratory FACE was perhaps most comparable, being a deciduous forest plantation with a high LAI of about 5.5, but the trees were younger and smaller, and the e[CO_2_] canopy volume was smaller and more uniform in each experimental patch (Norby et al., 2006).

FACE facilities have a simple scientific aim—that is, to subject ecosystem patches to consistent e[CO_2_]—but are complicated to engineer. In order to meet the science aim without altering other environmental parameters, the CO_2_ fumigation must be accomplished using infrastructure that minimally influences canopy structure, environmental aerodynamics and microclimate. BIFoR FACE consists of nine experimental patches of forest, three infrastructure arrays dosing air +CO_2_, three infrastructure arrays dosing with ambient air only and three noninfrastructure patches (see Section 2.2). Fumigation of 30 m diameter patches (see Table 1) is accomplished using approximately circular arrays of 16 free‐standing lattice towers, supporting perforated pipes from which premixed air/CO_2_ is released from the upwind quadrant (see Section 2.4).

**Table 1:** FACE array geometries

Array #	Array tower heights (m)	Internal radius (m)^a^	Research area (m^2^)	Volume (m^3^)	Central tower height (m)
1(f)	26.7	17	724	24,815	26.0
2(c)	25.6	16	628	21,107	24.9
3(c)	26.2	16	661	22,138	25.5
4(f)	27.2	17	702	24,406	26.5
5(c)	27.3	17	688	24,207	26.6
6(f)	24.7	17	678	21,641	24.0

a The internal radius is defined as the mean distance between the central tower and inside edge of the towers supporting the vent pipes. Arrays are designated fumigation ‘(f)’ or control ‘(c)’.

This paper presents an overview of the experimental infrastructure of the BIFoR FACE facility and examines the performance of the BIFoR FACE facility over its first growing season, with particular attention to factors affecting CO_2_ mixing ratios within and between arrays.

Within‐array spatial and temporal variability are potentially important factors in FACE projects because they determine the dose received by plants in each array. Detailed analyses have been made only once previously for a FACE system of similar size to BIFoR FACE: for a single array in an evergreen conifer plantation over two growing seasons (June–August 1994, May–October 1995; Hendrey et al., 1999). Similar analyses for smaller systems used, for example, on crop canopies, are not directly applicable, as they use pure CO_2_ injection, and variability in those latter systems is mostly analysed on a two‐dimensional basis (Mollah, Partington, & Fitzgerald, 2011). Hendrey et al. (1999) found that in the one array analysed in detail, FACE control was satisfactory within 90% of the entire volume, at least in the longer term (i.e. averaged over ~232 days). Hendrey et al. (1999) also found that CO_2_ consumption was positively related to wind speed and photosynthetically active radiation (PAR).

We analysed the full performance data of the facility for the first full season of fumigation in BIFoR FACE to address the following questions:

1. How does the enrichment achieved at the centre of the arrays vary over time?
2. To what extent is CO_2_ consumption in this deciduous forest ecosystem a function of PAR, wind speed and canopy phenology?
3. To what extent does CO_2_ release contaminate adjacent control areas?
4. How does the enrichment achieved vary throughout the canopy volume?

## 2 METHODS

### 2.1 Site description

The BIFoR FACE facility is located in central England (52.801°N, 2.301°W), United Kingdom. The facility is situated within a temperate, deciduous forest with *Corylus avellana* (common hazel) coppice underwood and with a *Quercus robur* (pedunculate oak) upper canopy that covers 19.1 ha. The forest consists of plant communities typical of a *Q. robur–Pteridium aquilinum–Rubus fruticosus* (W10a) and subcommunity *Holcus lanatus* (W10d) classification (Rodwell, 1991). The lowest point of the facility is at the site offices and CO_2_ storage plant is at +92 m a.s.l. The highest point is situated in the east of the forest, at approximately +112 m a.s.l. Areas of experimental interest are situated at +108 ± 2.7 m a.s.l. The dominant soil is Orthic Luvisol (FAO, 2015) with a mul‐moder humus classification. Underlying geology is a Helsby sandstone formation (BGS, 2018). Prior to securing the forest site, in the area where the 40 m flux tower is located, some timber trees were removed and the *C. avellana* understorey coppiced in early 2013 and so the area was not used when siting the FACE arrays.

**Figure 1:**
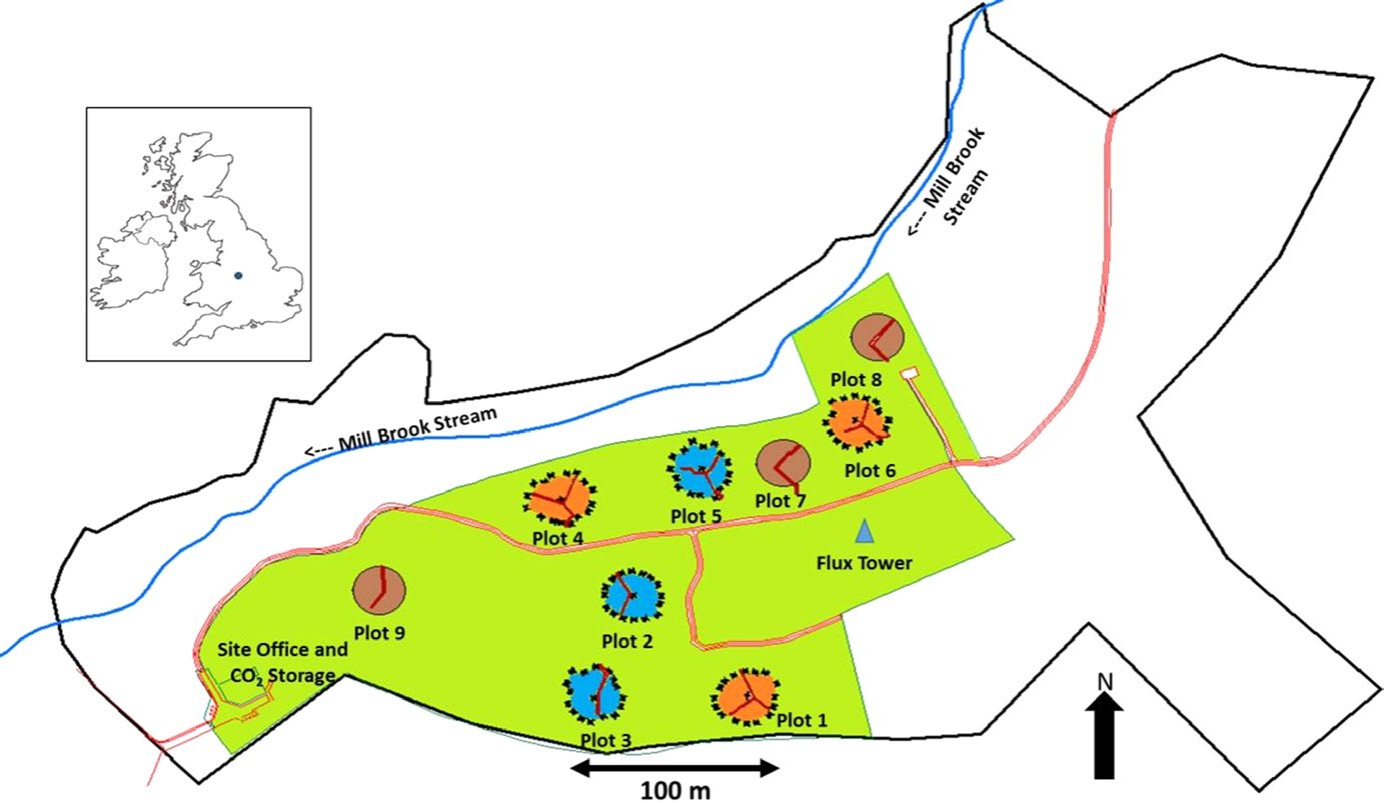
Schematic map of the BIFoR FACE facility within Mill Haft wood. Main access road highlighted in red. Mill Brook stream (blue line) passes through the northern periphery of the wood flowing NNE to WSW. Fumigated (e[CO_2_]) arrays highlighted in orange, infrastructure controls (delivering ambient air) are highlighted in blue, and noninfrastructure control arrays are highlighted in brown. All nine arrays contain elevated walkways, highlighted in red, to minimize footfall on the forest floor. Blue triangle denotes the position of a 40 m lattice tower (‘Flux Tower’) and location of an atmospheric sampling laboratory. Site office, CO_2_ storage and evaporation facility at bottom left next to the main entrance from the public highway. The green area represents the total experimental area controlled by the University of Birmingham covering 7.3 ha. The thick black line shows the border of the greater forest covering 19.1 ha. Inset map shows location in the UK

**Figure 2:**
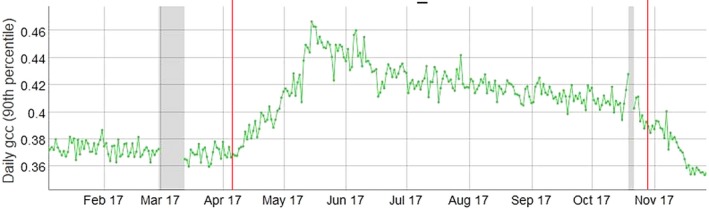
Canopy phenology at BIFoR FACE over 2017, within the field vision of the camera, shown as 90th percentile of the daily green chromatic coordinate (gcc) measurements. Seasonal switch‐on and ‐off times for the facility are indicated with red dashed vertical lines. Greyed periods indicate PhenoCam downtime due to technical problems. The PhenoCam is located on the ‘flux tower’ at 40 m height, looking west

The 2017 mean annual air temperature (T107 sensors, Campbell Scientific) at ~23 m height across the experimental site, was +10.3 ± 5.4°C (mean ± *SD*; using 1 min averages), about a degree warmer than the long‐term mean reported in Norby et al. (2016). The annual average air temperature across the experimental site during fumigation periods was only (i.e. daylight hours between April 4 and October 27, see Section 2.2) +14.5 ± 4.0°C (mean ± *SD*; using 1 min averages). The mean annual wind speed at the mature oak canopy (24–26 m height) was 2.2 ± 1.0 m/s (mean ± *SD*, temporal variation from the location of array 4 and calculated from 1 min averages). For annual air temperature, PAR, wind speeds and rainfall, see Figure S1. The dominant wind direction during the fumigation season was 215° (S/SW). The 2017 annual precipitation was 624 mm (measured by an ARG100 rain gauge by Campbell Scientific and located at an adjacent reference site; 52.807° N, 2.295° W), 66 mm below the mean annual precipitation reported in Norby et al. (2016). Canopy phenology was measured using a fixed position PhenoCam (Milliman, Hufkens, Richardson, & Aubrecht, 2017; Richardson, Hufkens, Milliman, & Aubrecht, 2017; Richardson et al., 2018; PhenoCam, 5MP, StarDot Technologies) located at the top of the 40 m lattice ‘flux tower’, facing south‐west towards arrays 1 (fumigated, f), 2 (control, c) and 3(c) (see Figure 1). The greenness index (gcc, Toomey et al., 2015) from the PhenoCam is shown in Figure 2 for 2017, which is typical of the four seasons to date for which we have greenness phenology data. A maximum gcc was observed on 15 May and minimum on 19 November, which corresponded to visual observations of canopy closure and leaf fall respectively.

### 2.2 Experimental set up

The BIFoR FACE facility consists of six infrastructure arrays (CO_2_ fumigated, *n* = 3; nonfumigated controls, *n* = 3), and three noninfrastructure arrays. Infrastructure arrays are paired so that a control (c) array will mimic the actions of its corresponding fumigated (f) array in real time, but only with ambient air. The pairings are numbered 1(f) and 3(c), 4(f) and 2(c), 6(f) and 5(c) (Figure 1). Array locations and pairings were determined during the early planning phase after baseline studies determined several forest characteristics (including soil analysis, plant area index, species distributions and biomass densities (MacKenzie et al., 2019). The FACE arrays operate up to 18 hr per day (05:00–22:00) between budburst to leaf fall, approximately 1 April to 1 November, depending on the solar angle (see Section 2.4 for more details). Scientific instrumentation and equipment is operational 24 hr per day and 365 days per year. Continuous measurements include wind speed/direction, PAR, total radiation, air temperature, humidity, barometric pressure, soil temperature, soil respiration, precipitation and ambient [CO_2_]. All research arrays have access to mains electrical power and connection to a fibre‐optic intranet. In addition to the FACE infrastructure, the site contains the flux tower; four meteorological masts at the forest edge; six array control buildings; office and site compound (Figure 1).

The operating target is an enhancement of [CO_2_] of +150 µmol/mol (150 parts per million by volume) above ambient at the top of the canopy at the centre of each treatment array (herein referred to as the ‘reference control port’, see Table 1 for tower heights). Deviations from the target value, in excess of ±30 µmol/mol (20%), are considered a control error. BIFoR FACE has employed a step change exposure (a ‘Square‐Wave’ exposure), similar to Duke FACE (He et al., 1996; Hendrey et al., 1999; Lewin, Hendrey, Nagy, & LaMorte, 1994), that commenced on 4 April 2017.

### 2.3 Array infrastructure

The design‐and‐build approach for the facility (2014–2016) was chosen to have minimal impact on the existing site. A similar approach was adopted whilst designing and constructing the FACE arrangement in the only other comparable experiment currently running (EucFACE, Hawkesbury Institute, Western Sydney University) (Drake et al., 2016; Ellsworth et al., 2017). However, significant differences in structural designs were adopted at BIFoR FACE.

To avoid bringing heavy machinery into the forest, screw piles provided foundations for all infrastructure, including site offices, towers and CO_2_ storage tanks. Screw piles were driven into the bedrock manually using air compression and emplaced grillages were then laser‐levelled to 0° slope. Array towers were bolted onto a level grillage platform, which provided a stable unguyed base, off the forest floor. Array towers were assembled from prefabricated galvanized steel. Complete array towers were individually winched into place using a specialist helicopter (Kamen K‐Max K‐1200, operated by Rotex Helicopter AG) which has very stable hover and low downdraft.

Each tower was inserted through the canopy onto the grillages to form the approximately circular FACE arrays. Array towers were sited in existing canopy gaps; the canopy directly above each tower location was surveyed using arboriculturists just prior to tower delivery to direct the pilot and remove or secure any hanging dead wood. Cutting of large branches was minimal and conducted only where siting of the tower was impeded. No dominant or subdominant trees were removed during the construction of the FACE arrays. CO_2_ storage and distribution to the arrays are described in the Supplementary Information.

The central towers provide canopy access to researchers and observers using a rope‐based canopy access system (CAS). The CAS comprises four cantilevers mounted onto the top of the central tower in all six infrastructure arrays, facing the four cardinal points of the compass and reaching 4 m outwards from the tower. A square‐rigged rope arrangement is anchored from the overhanging cantilevers and the tower base. A battery‐powered ascender (Harken Powerseat, Harken UK Ltd) is used to hoist personnel (sat in a bosun's chair) through the canopy for in situ research.

### 2.4 FACE arrays and experimental control

**Figure 3:**
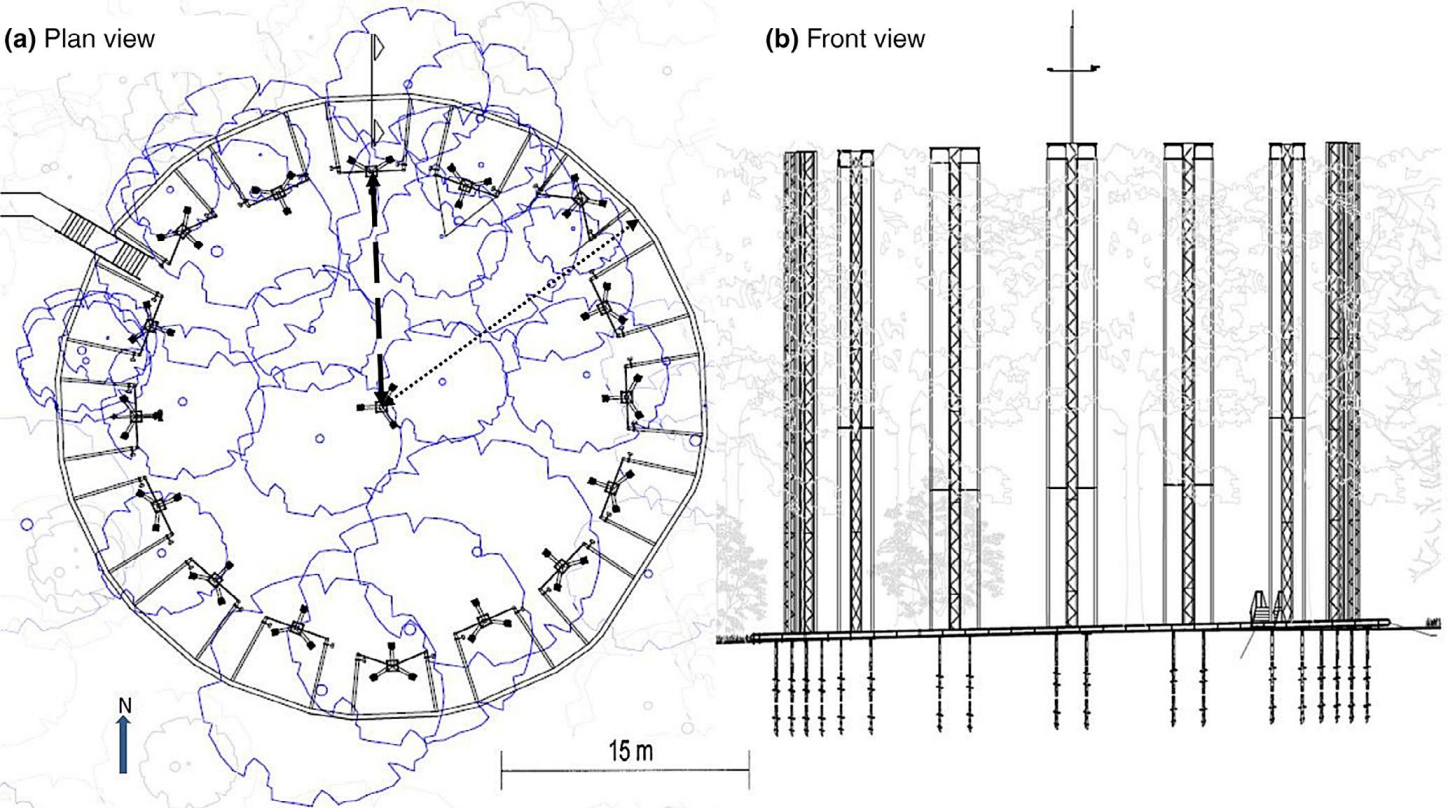
Example of a BIFoR FACE array. (a) Plan view of the structures encircling a 30 m diameter experimental patch. Dotted arrow shows radius from the centre to outer plenum edge (R ≈ 20 m) and the dashed arrow shows the radius from the centre to the internal edge of the research array (R ≈ 15 m). Note the nonuniform distribution of towers. This was due to the existing tree infrastructure (roots, trunk and canopy) that had to be avoided when installing the towers. (b) Front view showing screw pile system penetrating into the bedrock to provide secure anchoring and avoiding the need for guy cables to support the towers**.** The north tower is evident at the top of the plan view and in the centre of the front view, with a vertical pole containing a two‐dimensional ultrasonic anemometer (fumigated arrays only)

There are six FACE infrastructure arrays, each array comprising 16 peripheral towers with a central tower and a 15 m radius space for research. For separation distances between arrays, please see Table 2. Tower heights are designed to be ~1 m above the canopy height in each array and so vary between 24.7 and 27.3 m, as specified in Table 1. Towers are parallel in elevation, square in plan, and use an equilateral truss design (known as ‘Warren Truss’; Griggs, 2015). An example array plan and front elevation is presented in Figure 3. Each of the 16 peripheral towers in the array supports a pair of high‐density polyethylene vertical vent pipes (VVPs, 0.15 m internal diameter, *n* = 32 per array, Geberit UK), 2.95 m apart.

**Table 2:** Distance and direction matrix between FACE arrays

Array #	2(c)		3(c)		5(c)	
	Metres	Azimuth degree	Metres	Azimuth degree	Metres	Azimuth degree
1(f)	91	135	88	88	147	160
4(f)	74	310	128	356	83	253
6(f)	169	58	232	42	93	80

Note

Rows = fumigated arrays, Columns = control arrays. Direction is defined from the control array so that, for example, from the centre of array 2(c), the centre of array 1(f) is 91 m in direction 135° (south east).

e[CO_2_] is achieved by a computer controlled system that introduces varying volumes of pure CO_2_ gas into a fixed volume of ambient air (contained and circulating the periphery of the array inside a torus whilst being continuously replenished using an air intake fan), to a concentration of ~30 mmol/mol (3% by volume). The highly enriched air–CO_2_ mixture is then released from the VVPs in the upwind quadrant of the array. Process control uses a proportional‐integrative‐differential algorithm (Hendrey et al., 1999), to achieve a more uniform and efficient mixing effect within the treatment array (Lewin et al., 1994).

Ambient CO_2_ is measured using infrared gas analysers (IRGA, LiCor 840A, LiCor Lincoln) with inlets situated in the control arrays at ~24 m. The process‐control algorithm selects the lowest 1 min low‐pass filter average from the three control arrays and assigns this as the *set point* ([CO_2_]_set_) from which the treatment is calculated (i.e. ambient +150 µmol/mol). Analysers were calibrated every 2 weeks between 0 and 1,000 µmol/mol using ultrapure N_2_ (Air Liquide) and certified 1,000 µmol/mol CO_2_ in compressed air (Air Liquide) for the first 4 months, and monthly thereafter.

Daily fumigation times, on in the morning and off in the evening, were determined by solar elevation. This was calculated continuously by an FCP built‐in procedure based on Doggett (1987). The solar elevation used at BIFoR is −6.5° (roughly civil twilight). Predawn start up (i.e. at <0 degrees solar elevation), allows the arrays to attain the fumigation target before photosynthesis is significant. Array pairings start in sequence 1(f) + 3(c), 2(c) + 4(f) and 5(c) + 6(f) with a 5 min time lag. The planned operation times are, therefore, not exactly equal across the three pairings, varying by 51 hr over the season (Table 3).

**Table 3:** Planned operation hours, per array pairings of fumigation (f) and control (c), over the 2017 operating season

Array #	Planned operation time (hr)	Total operation time (hr)	Downtime (hr)	Daily average operation time (hr)	Minimum daily operation time (hr)	Maximum daily operation time (hr)
1(f) + 3(c)	3,108	3,032	76	14 ± 2.6	9	18
2(c) + 4(f)	3,075	2,992	83	14 ± 2.6	8	18
5(c) + 6(f)	3,057	2,973	84	14 ± 2.7	7	18

Note

Minimum operating hours vary by ~2 hr due to array‐specific engineering failures that were confined to single days of restricted running times. See Figure 1 for array locations.

The FACE Control Program (FCP) is designed to halt fumigation when canopy‐top, 1 min average, air temperature is <4°C, and resumes fumigation when the air temperature is ≥5°C. Carbon uptake under these conditions is considered negligible (Hughes, 1966).

Fumigation is also stopped during periods of high winds (15 min average wind speed, *u* > 8 m/s) due to the high cost of maintaining elevated [CO_2_] during windy conditions. Low wind speeds, *u* < 0.4 m/s, create conditions where advection of the highly enriched gas flow is ineffective. Under these conditions, gas is introduced all around the array via alternating VVPs (for more details, see Hendrey et al., 1999; Lewin et al., 1994). Dosing from alternating VVPs during periods of near‐still air avoids creating poorly mixed pools of air with different [CO_2_]. During normal conditions (0.4 ≤ *u* ≤ 8.0 m/s), eight upwind VVPs open to release the highly enriched air. Advection and turbulent mixing dilutes the highly enriched air to provide CO_2_ mixing ratios close to target in the centre of the array. Wind speeds are monitored using a two‐D ultrasonic anemometer (WMT700, Vaisala) ~1 m above the canopy on the northernmost tower of each treatment array.

### 2.5 Multiport sampling system

Lightweight lines strung from the central tower in each array to the peripheral towers support 32 air sampling tubes comprised of 0.064 m diameter (0.043 m internal diameter), black, UV‐resistant polypropylene tubing (Parker Hannifin) terminating in an inverted funnel (to prevent water ingress into the pipe). A sampling system with 32 solenoid valves under computer control sequentially samples each position on a 1 min time step. Measurements recorded for each inlet location consist of the average of thirty 1 s readings from a CO_2_ gas analyser (LI‐COR 840A, Lincoln). Each sampling line is purged for 30 s at 5 L/min before beginning each 30 s averaging period at 0.5 L/min. Sampling ports are located at the array centre at heights of 0.25, 2, 10, 14, 18, 22, 23–25 and 26–27 m (variable upper heights are due to differences in canopy height per array). To account for the spatial distribution across the volume of the array, additional sampling ports are located in a north–south, east–west cross pattern at 6 and 12 m horizontal distance from the array centre, at heights of 1, 10 and 25 m above the forest floor. This 32‐point system is installed in all six infrastructure arrays. For more details on the multiport sampling procedure, see Hendrey et al. (1999) and Lewin et al. (1994).

### 2.6 Statistical analyses and graphical applications

Data analysis and statistical calculations were conducted using a combination of Microsoft Excel 2013 and R (R Team, 2017; see also Bivand, Pebesma, & Gomez‐Rubio, 2013; Pebesma & Bivand, 2005). Pearson correlation coefficients report significance at thresholds of ***p < .001, **p < .01, *p < .05.

## 3 RESULTS AND DISCUSSION

### 3.1 Engineering performance

The first year of FACE operation started on 4 April (day‐of‐year 94) and lasted until 27 October (day 300) 2017, comprising 206 operating days. Start date was determined using current and previous years’ phenological observations taken at the site (Figure 2).

The facility‐average target was for 2,994 hr of operation (out of a total of 4,944 hr including night‐time). Based on previous work (Dodd et al., 2005; Hart, 1988; Hughes, 1966; Johnson et al., 1998), there was no CO_2_ fumigation for the 1,950 night‐time hours (i.e. solar elevation ≤−6.5°). Planned, actual and average daily operation times and total downtime per array are documented in Table 3.

The main FACE fumigation system was functionally operational for 2,928 hr (97.7% uptime) with ~66 hr of downtime due to ‘engineering faults’ (a term we use here to cover mechanical, CO_2_ supply, electrical and software issues) and wider environmental conditions (e.g. high winds and low temperature, see above). Over the operating season, a total of 17 hr were lost due to engineering faults or necessary infrastructure upgrades, accounting for 0.6% of downtime. These events were sometimes isolated to one FACE array at a time, allowing the rest of the facility to operate (Table 3). A national CO_2_ supply chain failure in early August 2017 resulted in 18 hr of engineering downtime for all three fumigated arrays, split over 2 days accounting for a further 0.6% downtime.

As discussed above, two main environmental considerations for FACE operation are wind speed and air temperature. Over the 2017 operating period, excessive wind speeds prevented operation for only 0.02% of the total operation time. Low air temperatures prevented fumigation start‐up in arrays 1(f) and 3(c) for a total of 32 hr, 31 hr in arrays 2(c) and 4(f) and 33 hr in arrays 5(c) and 6(f) (averaging 1.1% of total operation time). The low temperature events were largely confined to early mornings in April 2017.

A total of 4.76 × 10^6^ kg of liquid CO_2_ was delivered to BIFoR FACE in 2017. The site received 228 deliveries over the 2017 growing season (approximately 1 tanker load per day). Hourly CO_2_ consumption for the average of the three treatment arrays was 1,580 ± 657 kg CO_2_/hr (mean ± *SD*), or 23,500 ± 995 kg CO_2_/day, with minimum and maximum daily consumptions of 432 and 3,460 kg CO_2_/hr respectively. Average consumption is equivalent to 0.02 kg CO_2_ m^−3^ hr^−1^ of useful, fumigated air volume across the fumigated array volume and season.

Few e[CO_2_] experiments report CO_2_ consumption statistics for direct comparisons (Mollah, Edwards, Unwin, Fitzgerald, & Kilmister, 2017). Duke FACE (Hendrey et al., 1999) reported a daily consumption of 0.05 kg CO_2_ m^−3^ hr^−1^, with daily average wind speeds ranging between 0.7 to 3 m/s (with over half of the measurements having *u* < 1.3 m/s). Daily average wind speeds at BIFoR FACE ranged between 0.3 to 6.6 m/s, with only 21% of the measurements having *u* < 1.3 m/s. The ‘ETH FACE’ facility on meadow plants consumed on average 0.7 kg CO_2_ m^−3^ hr^−1^ (reported as 0.35 kg CO_2_ hr^−1^ m^−2^ and calculation determined from available data; Nagy, Blum, Hendrey, Koller, & Lewin, 1995). An open topped chamber experiment measuring the response of grape vines to e[CO_2_] had an average consumption of 0.06 kg CO_2_ m^−3^ hr^−1^ (median wind speed of 0.7 m/s; Mollah et al., 2017). These data indicate that the large‐scale BIFoR FACE is operating more efficiently than many predecessor facilities despite the higher wind speeds. This may be due to a denser canopy cover, slightly lower set point target than some of the experiments cited and a larger experimental volume which does not require constant replenishment as the CO_2_ has a longer residence time than in smaller scale experiments. The average values above suggest a mean residence time for additional CO_2_ in BIFoR FACE treatment arrays of 3.3 min.

A gradual decrease in demand was noted as the 2017 growing season progressed, with operation hours increasing from the April switch‐on until midsummer's day (24 June). Table 4 reports results from a correlation analysis of CO_2_ demand against five environmental variables. The analysis suggests that the primary driver of CO_2_ demand is wind speed explaining 21% of the variance. As wind speed increases, there is a clear requirement to provide more CO_2_ to maintain enrichment target (Table 5). PAR, gcc and air temperature all showed no or weak correlations with CO_2_ consumption. The actual amount of CO_2_ absorbed by the leaves is very small compared to the quantities released to maintain e[CO_2_] (Norby, Warren, Iversen, Medylen, & McMutrie, 2010).

**Table 4:** Pearson correlation coefficients for potential variables dictating daily total CO_2_ demand

	CO_2_	Wind speed	gcc	PAR	Temperature
CO_2_	1				
Wind speed	0.46***	1			
gcc	0.07	−0.24***	1		
PAR	−0.02	−0.05	−0.15*	1	
Temperature	−0.14*	−0.42***	0.38***	0.28***	1

Note

For explanatory variables, hourly averages were aggregated to determine a daily average for the hours of FACE operation.

Abbreviations: gcc, green chromatic coordinate; PAR, photosynthetically active radiation. Incrementing star symbols (*) represents the *p*‐values indicating the significance level of the correlation. In order, ***, *, to correspond with *p*‐values 0.001, 0.01, and 0.1.

**Table 5:** Summary statistics for daily total CO_2_ consumption for daily average wind speed categories

Daily CO_2_ consumption	Daily average wind speeds			
	<1 (m/s)	1 ≤ WS ≤2 (m/s)	2 > WS ≤3 (m/s)	3 > WS <5 (m/s)
Average (kg)	1.84 × 10^4^	2.27 × 10^4^	2.35 × 10^4^	2.43 × 10^4^
*SD* (kg)	5.47 × 10^3^	9.41 × 10^3^	1.02 × 10^4^	1.13 × 10^4^
Min (kg)	1.29 × 10^4^	6.60 × 10^3^	7.70 × 10^3^	6.50 × 10^3^
Max (kg)	2.42 × 10^4^	5.04 × 10^4^	5.46 × 10^4^	4.64 × 10^4^
Record count	5	80	88	34

Note

Daily wind speed data are calculated for periods of active fumigation. Record counts are whole days where the daily average wind speed fell into the wind speed category. There were no incidences where daily wind speed exceeded 4.7 m/s.

### 3.2 Experimental performance

**Figure 4:**
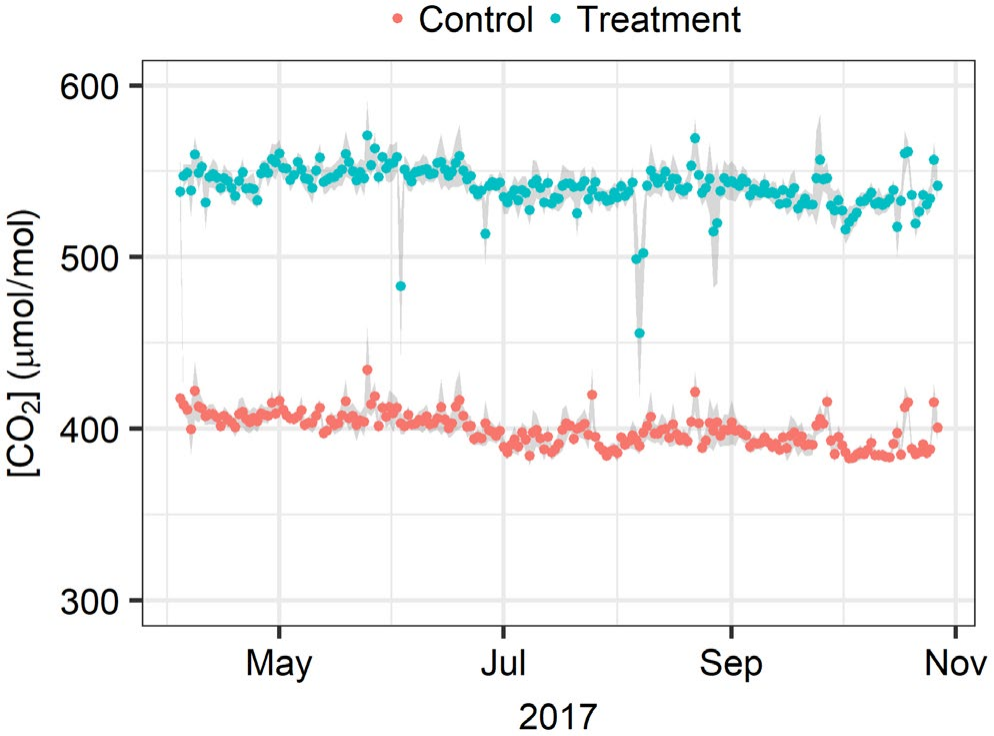
Seasonal course of daily average ambient [CO_2_] (red) and daily average elevated [CO_2_] (blue) at FACE array reference control ports. All data are taken from the 2017 fumigation period only (April 4–October 27) using the 1 min [CO_2_] averages. Grey areas show standard deviations between the three respective arrays that make up the treatment or control data sets. Data are shown for all times when the FACE system was scheduled to operate and, hence, includes all engineering failures and automatic shutdowns due to inclement weather

The enrichment set point (target) for the facility (+150 µmol/mol above ambient) is determined using a moving 5 min average, relative to the ambient concentration in the control arrays measured in real time and automatically fed into the FCP algorithm. To look at CO_2_ distributions in more stringent detail, statistics reported below are based on 1 min average [CO_2_] data, unless indicated otherwise. The ambient [CO_2_] over the season (during operating periods only), as determined using the control array ambient reference sampling ports, was 400 ± 17.0 µmol/mol (mean ± *SD*). The annual average enrichment value achieved was +147 ± 21 µmol/mol (mean ± *SD*, Figure 4). Table 6 reports the summary distribution statistics for the 1 min average [CO_2_] and the calculated enrichment value of the two treatment levels for all arrays.

**Table 6:** Summary distribution statistics for all arrays

e[CO_2_] µmol/mol											
Array	Average	*SD*	Skewness	Kurtosis	Q_1_	Q_5_	Q_25_	Median	Q_75_	Q_95_	Q_99_
1(f)	145	23	−3.0	18.9	21	115	140	148	155	168	185
4(f)	147	18	−4.4	34.9	65	127	142	148	155	166	178
6(f)	150	20	−2.1	39.9	53	130	145	151	158	173	186
Mean(f)	147	21	−3.0	29.2	26	125	142	149	156	169	184
2(c)	7	10	2.0	7.2	0	0	1	3	10	28	40
3(c)	4	6	5.7	92.1	0	0	0	1	4	14	21
5(c)	7	7	1.5	9.3	0	0	1	5	11	20	27
Mean(c)	6	8	2.5	16.7	0	0	1	3	9	21	34

Note

Mean, standard deviation (*SD*), skewness, kurtosis, and quantiles (Q1 (1%ile), Q5 (5%ile), Q25, median, Q75, Q95, Q99), reported as µmol/mol e[CO_2_] in comparison to the defined ambient signal using the 1 min [CO_2_] averages.

**Figure 5:**
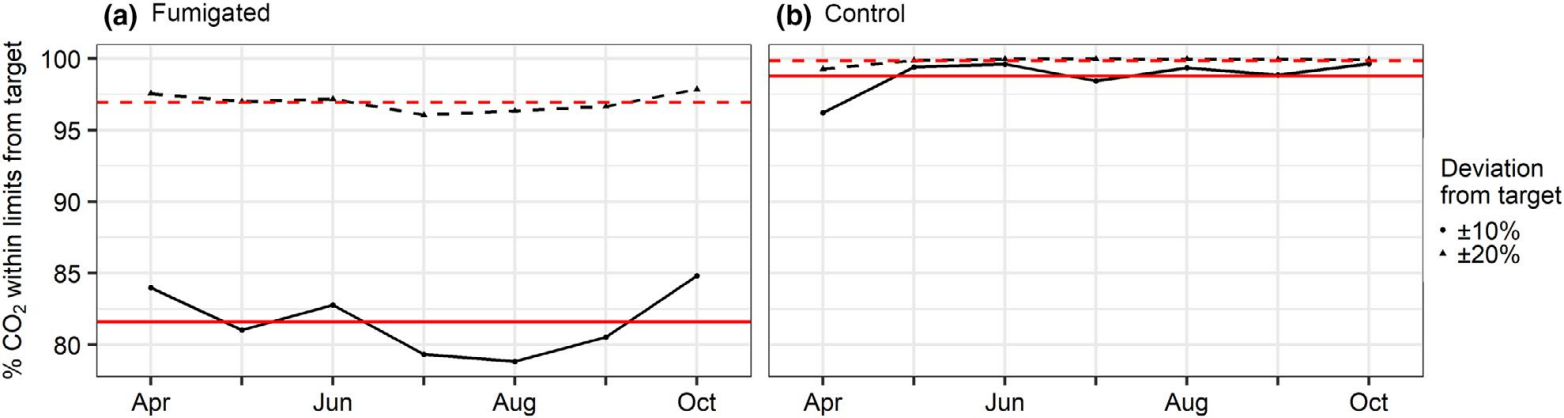
Deviations from averaged treatment and control targets. Measured against the a priori target for acceptable performance ±20% target e[CO_2_], for at least 80% operation time. (a) Fumigated arrays. (b) Control (ambient) arrays. Control array replicates were subject to variation from each other and the defined ambient target, mostly due to slightly different local conditions (stand density, canopy topography and respiration differences), time averaging differences of [CO_2_] measurements and wind‐borne cross contamination. Red‐dashed lines show the average measurement for the ±20% fumigation and control arrays across the season: Fumigation = 97%, Control = 100%. Solid red lines show the average for the ±10% fumigation and control array target: Fumigation = 82%, Control = 99%

In line with previous studies, we set the a priori goal for acceptable performance of the 1 min average e[CO_2_] to remain within ±20% of the set point, for at least 80% of the operation time (Hendrey et al., 1999; Miglietta et al., 2001). BIFoR FACE achieved its enrichment set point at 97% of the operation time across the three fumigated arrays, that is, well above target. A more stringent goal of being within ±10% of the enrichment target was achieved during 82% of the time (see Figure 5).

The averaged array performances shown in Figure 5 demonstrate the relative stability of the facility over the growing season. Ambient [CO_2_] in the three control arrays were always within ±20% of [CO_2_]_set_, and were within ±10% of [CO_2_]_set_ 99% of the time.

The average % deviation from target, using hourly averages of the 1 min enrichment data during fumigation periods, was +5 ± 14.5%, +7 ± 10.4% and +7 ± 12.1%, that is, not statistically different from zero, for arrays 1(f), 4(f) and 6(f) respectively. Across all the fumigation arrays, the deviation was 6 ± 12.4%, that is, also not statistically different from zero.

Figure S2 shows the monthly distributions of variance between the target (red line) and actual enrichment hourly averages for all hours when fumigation was scheduled to operate. Negative outliers represent events such as engineering failures (e.g. loss of CO_2_ feedstock in August); positive outliers represent calm weather events (wind speeds <0.4 m/s) resulting in short‐term over‐dosing.

There are three groups of outlier events (April, June and August) across the three fumigated arrays that indicate under performance was an issue at some point in those months (rather than short‐term deviations from the target. These were due to engineering shutdowns and all events have been catalogued (Hart, Miles, Harper, & MacKenzie, 2017). The cluster of negative outliers in August, for all three treatment arrays, was caused by a UK‐wide shortage of CO_2_ causing the facility to shut down operations due to low liquid storage levels. These shutdowns were managed to prevent fumigation shut down over an entire solar day and maximize fumigation exposure over the solar maxima. A critical shortage occurred on 6 August 2017, when the system was shutdown at 1200 hr and did not restart until 7 August at 0800 hr.

**Figure 6:**
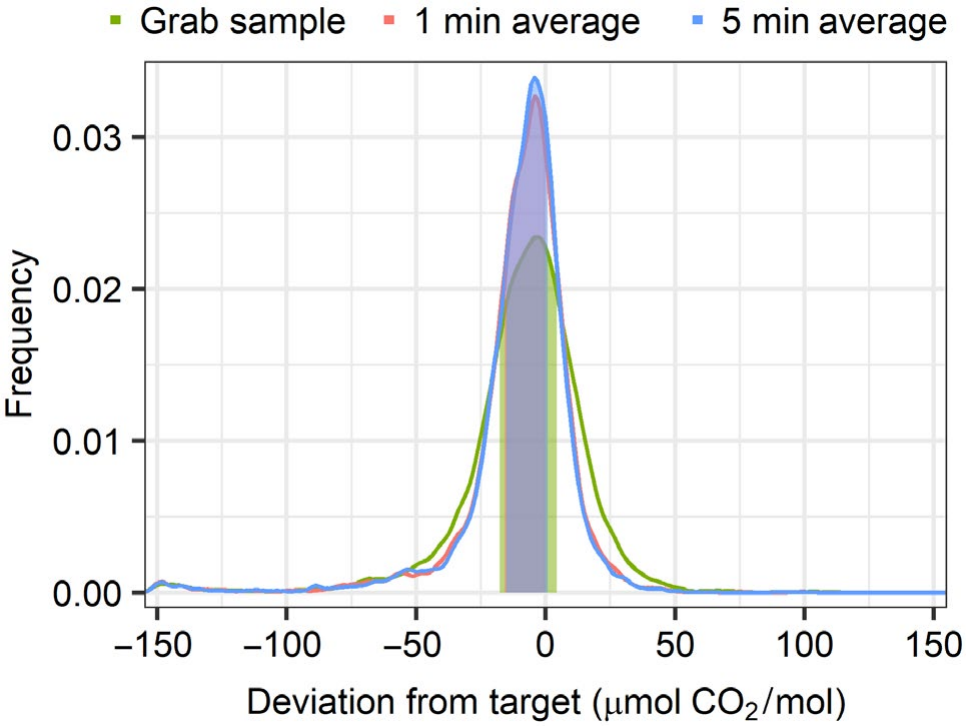
Kernel density plot of the facility average instantaneous grab, 1 min, and 5 min average [CO_2_] measurements at the control port (~24 m height) for the fumigated arrays over the seasonal operating period. The filled areas correspond to points between the 25th and 75th percentiles, see Table S2 for percentiles. Grab values are the instantaneous [CO_2_] measurements measured every 1 s; 1 min and 5 min averages are derived from the grab values as low‐pass filter averages

‘Grab’ e[CO_2_] samples are recorded as 4 s averages of 1 Hz measurements at the FACE array control ports. The 1 and 5 min averages are automatically calculated from these data to manage the facility and help assess its day‐to‐day performance (Figure 6). The grab e[CO_2_] data show larger excursions from the set point at both the low‐ and high ends of the distributions but with a clear positive skew, indicating a longer, higher tail for the high concentration side. Low‐end tails are capped when e[CO_2_] attains ambient conditions. Positive excursions indicate brief moments when e[CO_2_] overshot the target, in some rare cases by up to +390 µmol/mol above ambient. The grab, 1 min, and 5 min enrichment averages are very similar, the distribution of 5 min averages having a higher peak and slightly narrower width. The density distribution is centred below zero, indicating that the system was generally slightly below target.

### 3.3 Cross contamination of control arrays

It is important to quantify the level of cross contamination between fumigated and control arrays (e[CO_2_] fumigation—e[CO_2_] control). Control array contamination from advected e[CO_2_] reduces the effective dose in BIFoR FACE treatment arrays. Control arrays were measured at the same sampling rate and position within the upper canopy to determine deviations from [CO_2_]_set_, which is defined as the lowest [CO_2_] measured across the three control arrays:that is, Δ[CO_2_] = [CO_2_]_control_ − [CO_2_]_set_. Deviations are observable for the month of April with respect to both the 10% and 20% targets (Figure 5). For example, strong cross winds in April led to Δ[CO_2_] in array 2(c) exceeding the ±10% threshold for 8% of the time. Norby et al. (2006) reported an average contamination of 10 µmol/mol CO_2_ for the Oak Ridge FACE facility.

**Figure 7:**
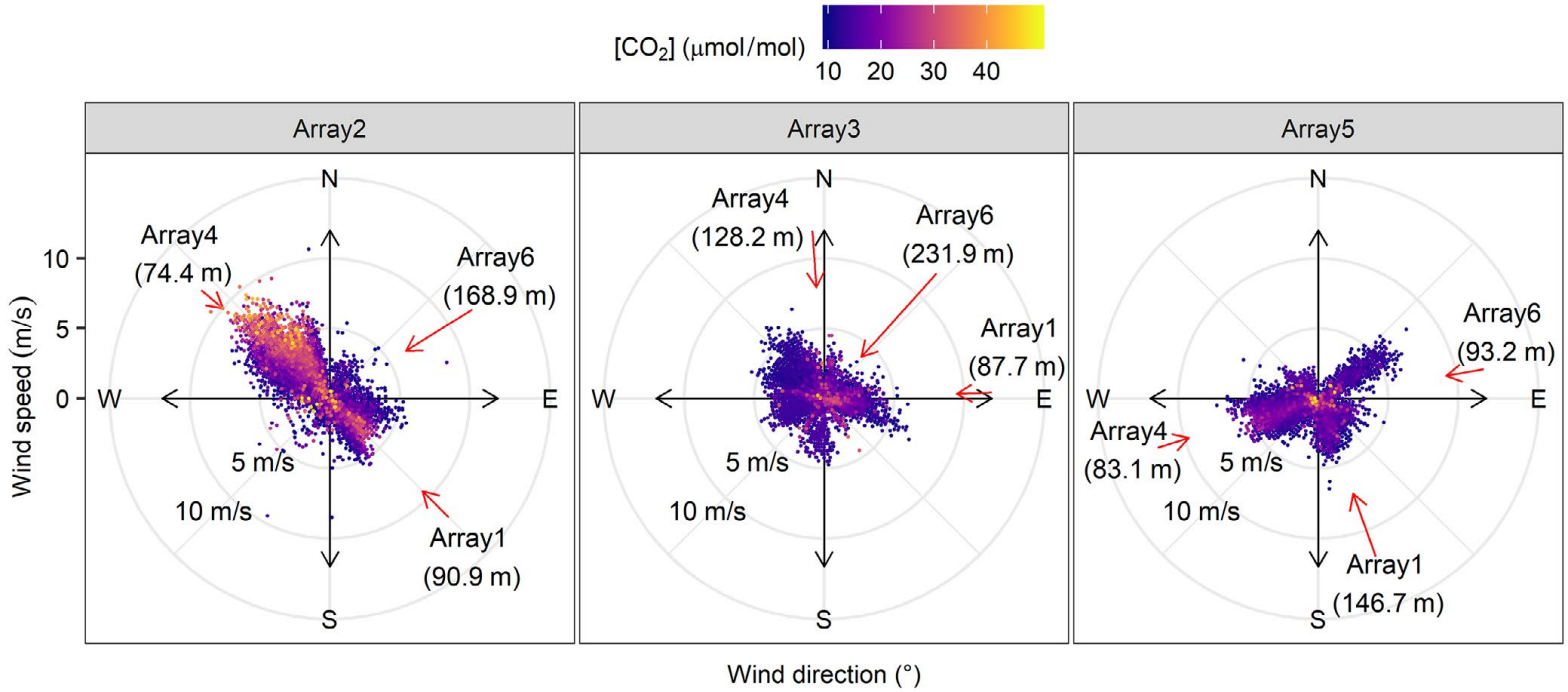
Wind speed and direction for 5 min averages for instances when Δ[CO_2_] ≥ 10 µmol/mol occurred in control arrays, relative to the facility reference control set point, [CO_2_]_set_. Data points represent enrichment values with respect to [CO_2_]_set_ and are sorted by taking the mean value for each 1° of cardinal direction and for each wind speed increment of 0.25 m/s. Distance and direction to the fumigated arrays are denoted by red arrows. Length of the arrow indicates distance between arrays (see Table 2 for measured distance and azimuth direction matrix)

The degree of cross contamination is shown in Figure 7 for those occasions when 5 min average of [CO_2_]_control_ ≥ 10 µmol/mol with respect to the reference control port. Changes in wind direction, other than south and south‐westerly, had the largest impact on [CO_2_]_control_. Increasing wind speeds also enhanced contamination when in combination with directional changes away from dominant south‐westerlies. However, low winds (≤5 m/s) account for the majority of events. The number of contamination events was 44,807, 25,724 and 54,711 for arrays 2(c), 3(c) and 5(c) respectively. This equates to 747, 429 and 912 hr for which the control arrays were subject to modest contamination (Δ[CO_2_] > 10 µmol/mol) for arrays 2(c), 3(c) and 5(c) respectively, which is equivalent to 23%, 15% and 30% of the actual operation time.

These contamination data reflect the array positions within the forest (Figure 1) and the proximity of neighbouring treatment arrays in relation to fluctuations in wind direction. Array 5(c) had the highest number of observable incidences and is positioned between fumigated array 4(f) (83.1 m distant) to the west and fumigated array 6(f) to the East (93.2 m distant). Therefore, any easterly or westerly deviations in wind direction, from the typically south‐westerlies, result in modest cross contamination (Figure 7).

**Figure 8:**
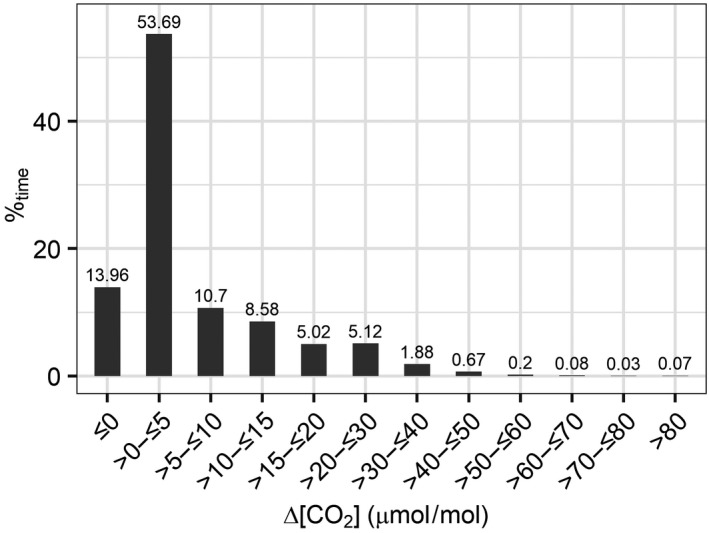
Histogram of cross‐contamination incidents in all control arrays (2(c), 3(c) and 5(c)). Δ[CO_2_] is defined in the main text

Figure 8 shows a histogram of 1 min average Δ[CO_2_] from the control arrays, when falling within the cross‐contamination criterion. The figure shows a strong mode at 0–5 μmol/mol. Note that the reference [CO_2_]_set_ is a low‐pass filter 1 min average, so it is possible to have 1 min averages of Δ[CO_2_] < 0 µmol/mol. Incidences of cross‐contamination with Δ[CO_2_] > 80 µmol/mol accounted for less than 0.1% of the enrichment period. Contamination events with 0 < Δ[CO_2_] ≤ 15 µmol/mol accounted for 73% of the occasions when [CO_2_]_control_ were flagged as contaminated. Incidents with Δ[CO_2_] < 0 µmol/mol accounted for 14% of the time and minima ranged between 0 and −11 ± 3.7 µmol/mol (mean ± *SD*) averaged across the three control arrays.

These data would suggest that CO_2_ released from the fumigated arrays may, very occasionally, travel several tens of metres in or over the canopy, depending on wind speed and direction. Figure 8 demonstrates that the rare contamination events in the control arrays are attributable to point sources (fumigation arrays).

### 3.4 Three‐dimensional [CO_2_] fields inside the arrays

**Figure 9:**
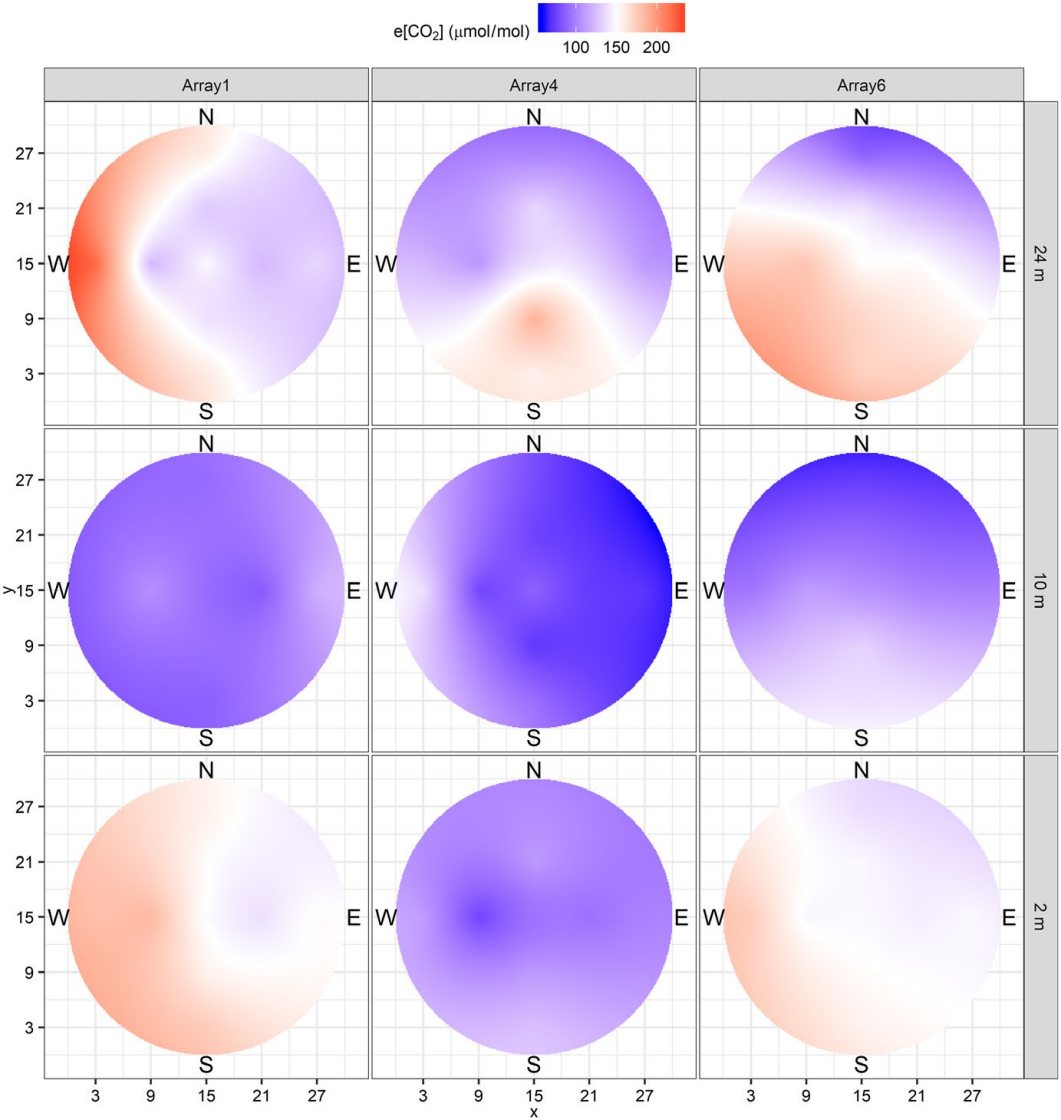
Interpolated mean distribution of e[CO_2_] (µmol/mol) in the fumigated arrays for the 2017 season measured at three heights (m) from the ground (2, 10 and 24 m). *X* and *Y* axes units are meters (m) from the internal array edge. Kriging was performed after fitting a variogram using a linear model with partial sill = 1 and range = 0. The measurements informing this interpolated field are at 12, 6 and 0 m from array centre in the four cardinal compass directions

The multiport samplers allow for a more detailed analysis of the spatial distribution of the [CO_2_] within each array (see Section 2.5). For consistency, only ports at the same heights were included in this analysis. It takes 32 min to poll all 32 inlets (providing ~2 measurements per hour per port), during which time the flow of CO_2_ into the array will be adjusted many times by the FCP, so measurements are not instantaneous snapshots of the three‐dimensional field. Figure 9 shows season‐average height ‘slices’ through the array, using only daytime data collected during fumigation, spatially interpolated using kriging and linear fitting of the variogram.

CO_2_ mixing ratios are higher on the 2 and 24 m horizontal planes than on the 10 m plane. Red to blue areas in Figure 9 denote fixed sampling positions where e[CO_2_] were consistently high or low (compared to the target concentration) across the season, presumably as a result of imperfect mixing in the lee of a tree stem. e[CO_2_] was higher in the south–west quadrant for all three arrays, corresponding to the prevailing winds.

Detailed performance statistics for each of the analysed cross sections are provided in Table S3. To summarize: at 24 m, the e[CO_2_] field was 100 ± 19%, 88 ± 8% and 101 ± 25% of the target for Arrays 1(f), 4(f) and 6(f) respectively. For array 1(f), eight of the nine inlets were within ±20% of the operating target over the seasonal average. All nine inlets were within ±50% of the target. The high enrichment deviations that are clustered between the west and north perimeters are likely caused by the position of the array on the south‐east edge of the forest. For array 4(f), five of the nine inlets were within ±20% of the operating target over the seasonal average. All nine inlets were within ±50% of the target. For array 6(f), eight of the nine inlets were within ±20% of the operating target over the seasonal average. All nine inlets were within ±50% of the target.

At 10 m, the e[CO_2_] field was 64 ± 10%, 56 ± 7% and 70 ± 16% of the target for Arrays 1(f), 4(f) and 6(f) respectively. For array 1(f), none of the nine inlets were within ±20% of the operating target over the season. All nine inlets were within ±50% of the target. For array 4(f), one of the nine inlets was within ±20% of the operating target. Five of the nine inlets were within ±50% of the target. For array 6(f), two of the nine inlets were within ±20% of the operating target. Eight of the nine inlets were within ±50% of the target.

At 2 m, the e[CO_2_] field was 107 ± 16%, 70 ± 7% and 100 ± 16% of the target [CO_2_] for arrays 1(f), 4(f) and 6(f) respectively. For array 1(f), eight of the nine inlets were within ±20% of the operating target over the season. All nine inlets were within ±50% of the target. For array 4(f), one of the nine inlets was within ±20% of the operating target. All nine inlets were within ±50% of the target. For array 6(f), all nine of the inlets were within ±20% of the operating target.

There is a clear tendency for the air just below the main *Q. robur* canopy (~10 m height) to have the lowest e[CO_2_], which reflects a design criterion when setting up the fumigation system. To maximize the exposure of the upper canopy trees to e[CO_2_], and to ensure enough back pressure is available to transport CO_2_ enriched air up the VVPs, many of the available outlet ports on the VVPs have been closed. It may be possible in future seasons to change which outlets on the VVP are open in order to improve the performance at 10 m without degrading performance at 25 m.

Lower levels of e[CO_2_] were observed in array 4(f) across the three different levels. This array contains the lowest amount of physical biomass (MacKenzie et al., 2019), and experiences the highest average winds speeds, but also, received the lowest mass of CO_2_ gas over the 2017 season. Array 4(f) operated at 98% of the target (i.e. 3 µmol/mol CO_2_ below target) across the season (Table 6, above).

**Figure 10:**
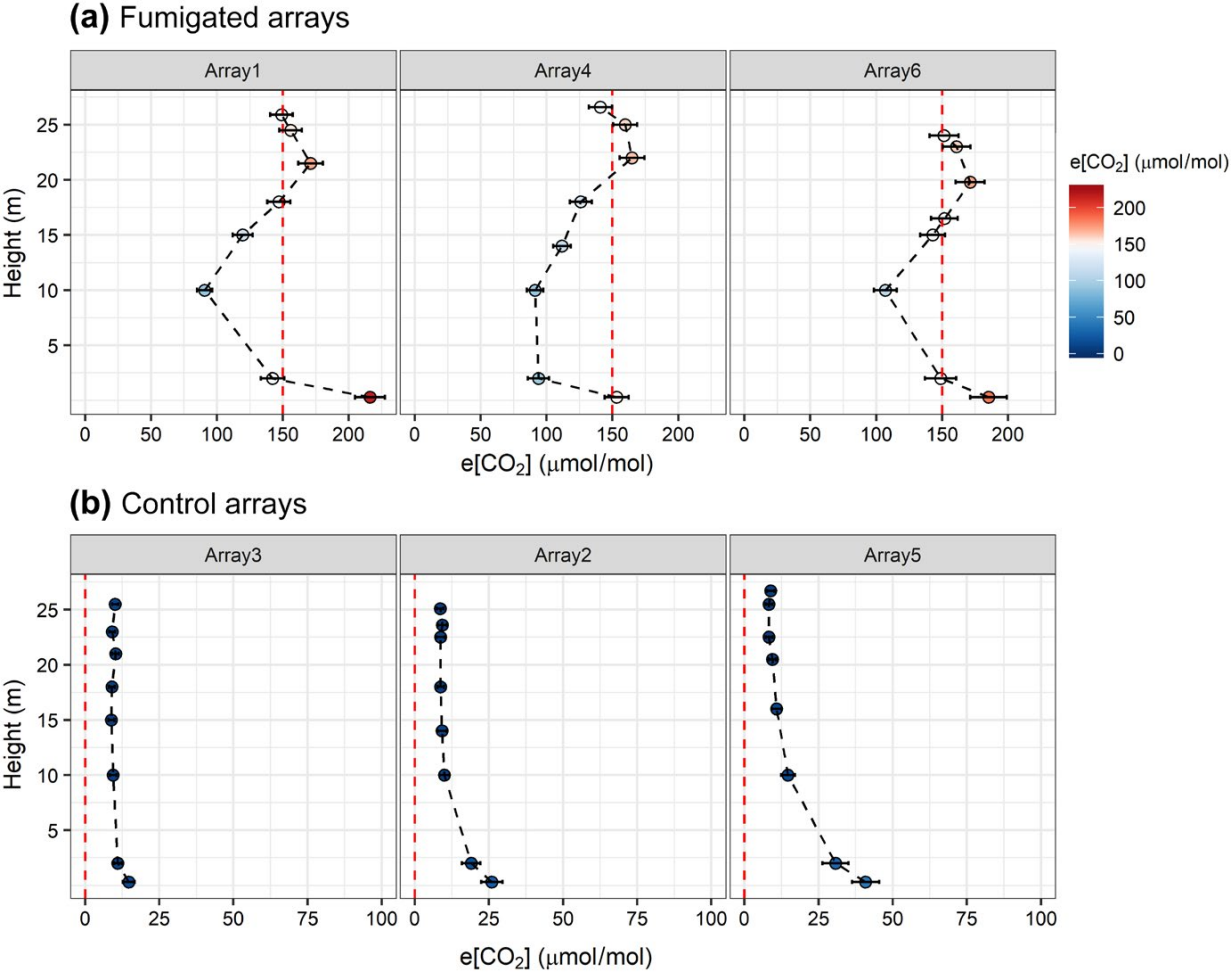
(a) Season‐average vertical profiles of Δ[CO_2_] for the treatment arrays (1(f), 4(f) and 6(f)), measured at the central tower of each array. The target concentration of +150 µmol/mol is denoted by the vertical red‐dashed line. (b) Season‐average vertical profiles of Δ[CO_2_] for the control arrays (2(c), 3(c) and 5(c)). Note that x‐axis scales are different between (a) and (b), and that the sampling heights are not exactly the same between arrays because measurements are relative to canopy top rather than height above ground

A vertical profile of the Δ[CO_2_] in the fumigation and control arrays are measured using eight fixed points at the centre of each array (Figure 10). The seasonal Δ[CO_2_] vertical profiles for the treatment arrays (measured from just above ground level to above canopy) show a nonuniform distribution (Figure 10a). The red dashed line in Figure 10a shows the target set point that should be achieved at each height. However, a visual survey in each array determined that there was minimal vegetation between the top of the coppice canopy (~8 m) and the base of the dominant oak canopy (~15 m). Therefore, only five of the 15 available outlet ports were opened to release CO_2_ enriched air between those heights. Therefore, the lower Δ[CO_2_] at 10–15 m is not unexpected, but should have only minor implications for the facility as very little actively photosynthesizing material exists at these heights. Outlet ports are arranged closer together and increase in number above ~14 m in order to distribute CO_2_ enriched air more widely across the *Q. robur* canopy (lower, middle and upper canopy). For heights above 18 ± 3 m, the Δ[CO_2_] was 99%, 99% and 104% of the enrichment target, for arrays 1(f), 4(f) and 6(f) respectively (Figure 10). These statistics agree with the single‐point FCP data discussed in Section 3.2 and demonstrate that the entire upper storey canopy is being adequately enriched.

For 0.25 < height < 10 m, Δ[CO_2_] was 78%, 62% and 85% of target, for arrays 1(f), 4(f) and 6(f) respectively. These results indicate that the coppice canopy and understorey were exposed to a significant step change in [CO_2_]. Near the ground (0.25 ≤ height ≤ 2 m), there was high variability in Δ[CO_2_] between arrays and between the two measurement points. The centre of the fumigated arrays was overdosed at the ground level (as defined by the enrichment target) by 144%, 123% and 131% for arrays 1(f), 4(f) and 6(f) respectively. The 0.25 m position showed high Δ[CO_2_] which was likely a combination of soil respiration and CO_2_ enriched air travelling into the centre location. Comparisons to the control arrays (Figure 10b) indicate that soil/root respiration had an appreciable influence near the ground (cf. Schlesinger & Andrews, 2000).

The vertical profiles of Δ[CO_2_] observed in the control arrays (Figure 10b) demonstrate a clear increase in [CO_2_] between 0.25 and 2 m. Deviations from [CO_2_]_set_ at 0.25 m were 26 ± 6, 14 ± 1 and 36 ± 17 µmol/mol for arrays 2(c), 3(c) and 5(c) respectively. This indicates the positive impact of soil and leaf litter respiration to lower strata of the local atmosphere within the arrays. The magnitude of soil respiration is site dependent and varies with environmental parameters including soil moisture, vegetation coverage, site elevation and substrate quality (Marconi, Chiti, Nolè, Valentini, & Collalti, 2017; Rustad, Huntingdon, & Boone, 2000). From 10 m, CO_2_ is well‐mixed and close to [CO_2_]_set_. Δ[CO_2_] is small and positive because [CO_2_]_set_ is defined as the minimum observed in any control array.

The spatial distribution analysis presented here has implications for the area of each array that is suitable for research. When discussing FACE array sizes, two diameters are often mentioned in the literature, and are sometimes incorrectly interchanged. These are: (a) the diameter of the circle of pipes emitting CO_2_; and (b) the diameter of useful experimental ‘real estate’. It is important to understand the differences between these diameters when locating experiments within a FACE array and when calculating array areas and volumes. As the measurement field within the array infrastructure only commences 3 m away from the encircling emitter pipes, it is recommended that experiments concerned with e[CO_2_] restrict themselves to remain within the internal multiport measurement boundary that is, maximum of 13 m from the array centre.

## 4 CONCLUSIONS

The Birmingham Institute of Forest Research Free‐Air CO_2_ Enrichment (BIFoR FACE) facility has been built into an established temperate deciduous forest. The infrastructure has been built without concrete foundations, which would have resulted in significant changes to the soil structure, without guy wires, which would have required removal of overhanging branches and considerable numbers of ground anchors (causing further soil disturbance), and without significant change in canopy cover. One hundred and two 25 m tall, metal lattice infrastructure towers are sited in existing forest gaps and are supported by manually inserted screw piles. e[CO_2_] began in April 2017 and is scheduled to continue until 2026. Addressing the research questions posed above, we find the following.

1. How does the enrichment achieved at the centre of the arrays vary over time?

On analysing e[CO_2_] throughout the 2017 growing season, it was determined that the BIFoR FACE facility has exceeded its design targets. The grand average free‐air e[CO_2_] was +147 ± 21 µmol/mol, as given by 1 min average [CO_2_] measured at the top of the canopy in the centre of the three treatment arrays. The grand average perturbation to ambient [CO_2_] in the three control arrays was not significantly different from zero (+6 ± 8 µmol/mol), with respect to an ambient set point defined as the lowest 1 min low‐pass filter average amongst the control array measurements. The treatment arrays were within 10% (15 µmol/mol) of target for 81.6% of scheduled operation time, and within 20% of target for 96.7% of scheduled operation time. Deviations from the enrichment target were predominantly due to engineering and CO_2_ supply issues.

- To what extent is CO_2_ consumption in this deciduous forest ecosystem a function of PAR, wind speed and canopy phenology?

For its first growing season of e[CO_2_], comprising just under 3,000 hr of operation, BIFoR FACE required 4,760 tonnes of CO_2_. Wind speed explained 21% of the variance in CO_2_ demand; PAR and temperature did not significantly affect CO_2_ demand, although both are used to define thresholds for pausing the CO_2_ enrichment.

- To what extent does CO_2_ release contaminate adjacent control areas?

Contamination of the ambient control arrays by e[CO_2_] from the treatment arrays is rare and short‐lived, being mostly governed by short periods when winds shift away from the predominant south‐westerlies. Control arrays are within 10% of the ambient set point—defined as the lowest 1 min low‐pass filter average amongst the control array measurements—98.8% of the time. When contamination does occur, only 13% of such events produce 1 min average [CO_2_] perturbations in excess of 15 µmol/mol. Array 2(c) experienced more frequent cross contamination events, but of a much lower intensity, than control arrays 3(c) and 5(c). Array 5(c) suffered more extreme enrichment events, with treatment array 6(f) providing the majority of the source.

- How does the enrichment achieved vary throughout the canopy volume?

We have captured what we believe to be the most comprehensive data on the three‐dimensional distribution of [CO_2_] in a forest FACE facility. Each array contains 32 gas‐sampling inlets, placed at the array centre and at 6 and 12 m distance in each of the cardinal compass directions, at approximately 2, 10 and 24 m above ground. For operational reasons, [CO_2_] tend to be lower at 10 m height than above or below. The median [CO_2_] values in the reconstructed [CO_2_] fields show enrichment lower than the target but still well above ambient.

We continue to monitor performance of the facility overall, and to make measurements of [CO_2_] with high temporal and spatial frequency in the arrays allowing for a more detailed assessment of the three‐dimensional FACE statistics. This will also be a valuable ‘tracer’ data set to derive forest canopy turbulence and mixing statistics, particularly when combined with sonic anemometer data from instruments within and around the BIFoR FACE forest patch.

Based upon the facility design and the continuous monitoring of the engineering control systems, in line with the local environmental conditions, BIFoR FACE operated within its design parameters for the majority of 2017. BIFoR FACE has demonstrated over its first operation season that it will provide an extensive, consistent and reliable data set for the analysis of e[CO_2_] in a seminatural, temperate, deciduous, mature forest. These data, and ongoing sample collections, will provide an essential resource for modelling the potential impacts and effects of e[CO_2_] on other similar landscapes.

## CONFLICT OF INTEREST

All listed authors declare no conflict of interest.
