# Supplementary material for: Characteristics of free air carbon dioxide enrichment of a northern temperate mature forest
Source: Glob Chang Biol. 2019 Sep 11;26(2):1023–37. doi: 10.1111/gcb.14786 (PMC7027798; doi:10.1111/gcb.14786)
Supplement: Supplementary file 1 [file GCB-26-1023-s001.docx]

**Supplementary Information**


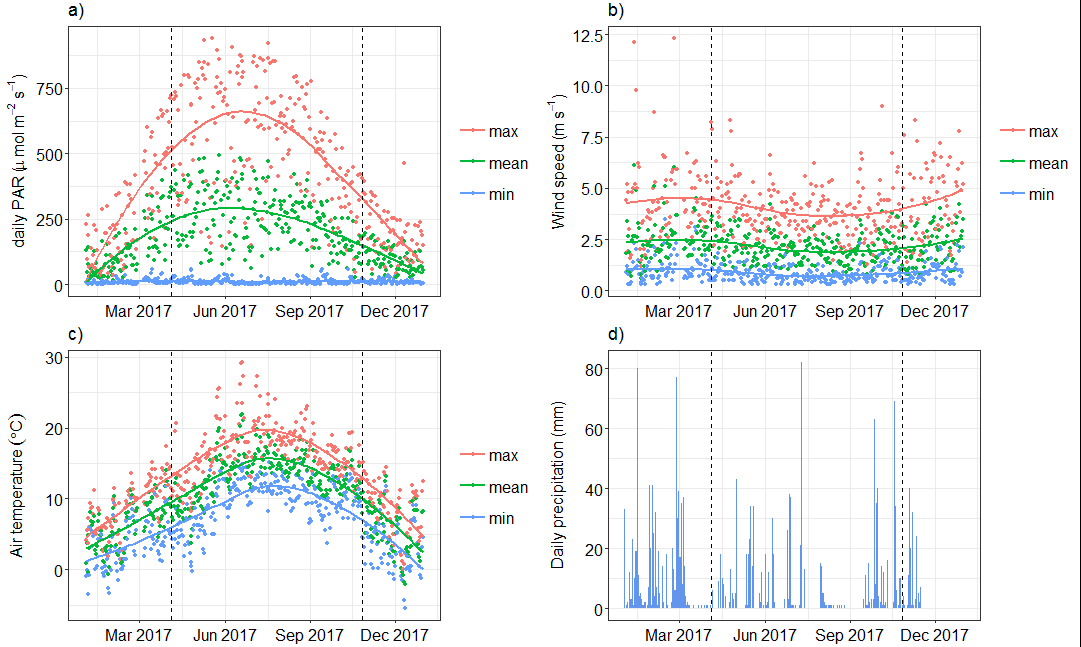


**Figure S1**: Annual ambient daily environmental conditions for the BIFoR FACE site, Mill Haft, Staffordshire, UK, 2017 operating period (vertical dashed lines denote the fumigation operating period). a) Seasonal course of daily mean, minimum and maximum PAR, with 0 values excluded to demonstrate true solar minimum values before sunset, b) daily mean, minimum and maximum air temperature, c) daily mean, minimum and maximum wind speeds and d) daily precipitation totals. Variables in (a)-(c) measured at the top canopy level (~25 m) across three FACE arrays, precipitation (d) measured at ground level in an open field adjacent to Mill Haft.


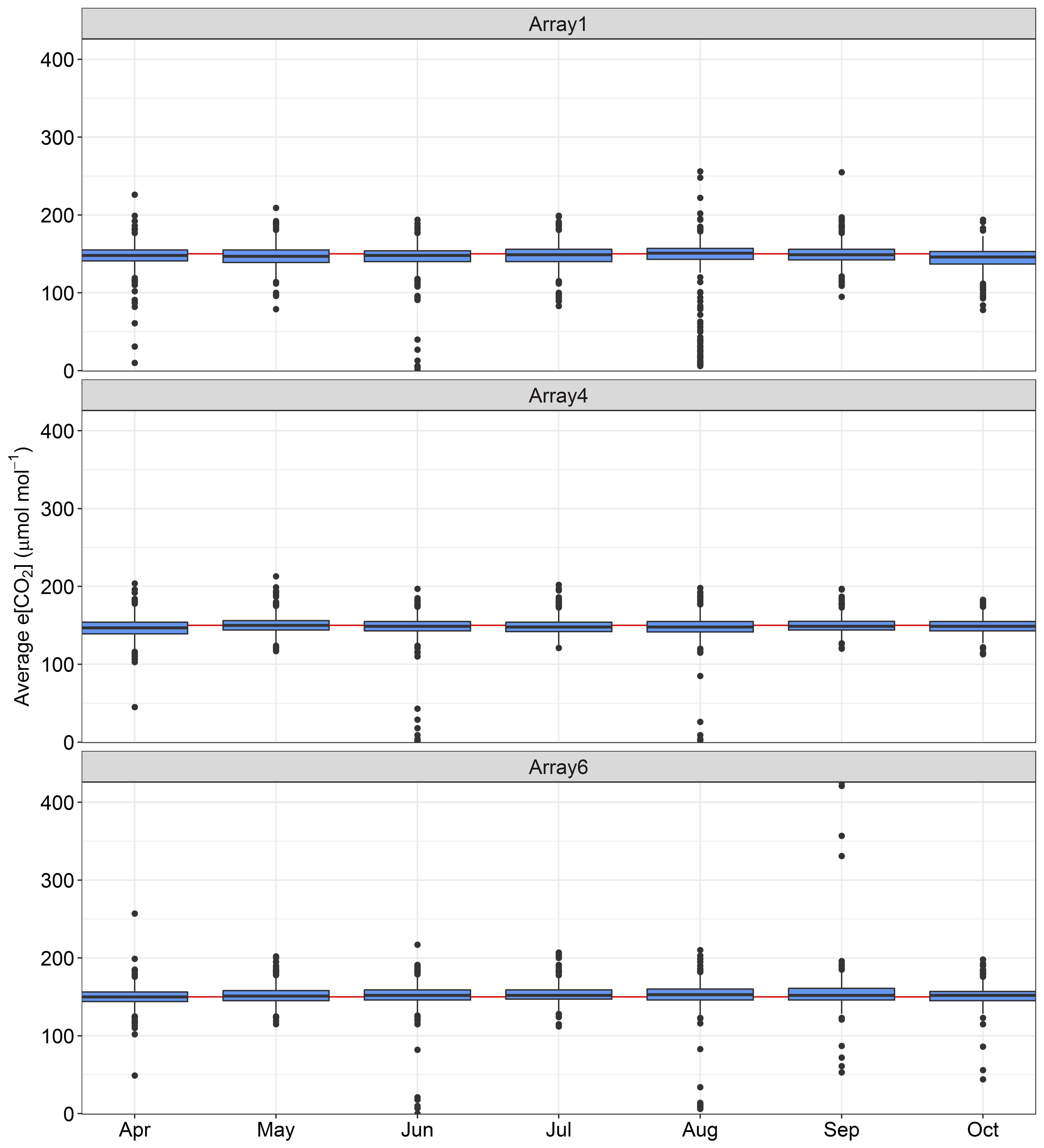


**Figure S2: Figure S2**: Monthly distributions of array performance against the set point target of + 150 µmol mol^‑1^ CO_2_. Statistics calculated using the 1‑minute averages to determine the daily average of the reference control port (~23‑25 m, depending on array) located at the centre of the research array. All data includes engineering failures and excludes non-fumigation periods (e.g. night). Dots plotted on these box-and-whisker plots show individual data points outside 1.5 times the inter-quartile range.

*CO_2_ Storage & Distribution*

Liquefied CO_2_ is sourced as a by-product from agricultural fertiliser production. Food grade CO_2_ was transported to BIFoR FACE by road trucks in ~20,000 kg loads (average delivery of 21,453 ± 2,762 kg) once-to-three-times per day. CO_2_ was contained in vacuum insulated tanks (n = 3) with a maximum holding capacity of 1.4 x 10^5^ kg. Tank pressure was maintained at 1800 kPa and -20 ^o^C to keep CO_2_ in the liquid phase. An ambient air evaporator maintained this pressure regardless of demand for CO_2_ by the FCP. During fumigation, liquid CO_2_ was supplied to forced-air evaporators which vaporised the CO_2_ as needed. After evaporation the gas was then heated by an electric super-heater to a set point of 80 ^o^C. The gaseous CO_2_ was transported into the forest through a series of insulated, 10 cm internal diameter, stainless steel pipes (totalling 648.9 m). The temperature was reduced at stages (via adiabatic expansion at pressure reduction valves) to ~35 ^o^C, and pressure reduced to 250 kPa. CO_2_ dosage flow was measured at the array control shed by an electronic flow sensor and throttled by a rotary ramp metering valve (Model 735, Kurz Instruments, Monterey, CA). The metering valve provided an even, linear gradation of gas flow over the range 0–2000 kg h^–1^ and has been used in previous forest FACE facilities (e.g. DUKE FACE and EucFACE). The metering valve was operated directly by the bespoke FACE Control Program (FCP) software and firmware developed by Brookhaven National Laboratories (Hendrey et al., 1999). CO_2_ gas was then released into a torus-shaped plenum immediately down-stream of the constant air supply fan to be pre-diluted and mixed before introduction into the cylindrical air space of the array.

**Table** **S1**: Wind direction analysis of array 2(c), where [CO_2_] greater than the defined ambient target was determined to be artificially elevated. Data representative of April, 2017 only at BIFoR FACE.

| Cardinal Direction | Degree Direction | | Average CO_2_ (µmol mol^-1^) | Stdev  (µmol mol^-1^) | Average WS (m s^-1^) | Stdev  (m s^-1^) | Time Elevated (minute) | Time Elevated (Hour) |
| --- | --- | --- | --- | --- | --- | --- | --- | --- |
|  | **Start** | **Finish** |  |  |  |  |  |  |
| N | 348.75 | 11.25 | 29.1 | 13.9 | 2.3 | 1.5 | 182 | 3.0 |
| NNE | 11.26 | 33.75 | 22.3 | 8.8 | 2.0 | 0.9 | 57 | 1.0 |
| NE | 33.76 | 56.25 | 25.2 | 10.7 | 1.5 | 0.7 | 67 | 1.1 |
| ENE | 56.26 | 78.75 | 22.8 | 8.4 | 1.5 | 1.0 | 28 | 0.5 |
| E | 78.76 | 101.25 | 20.7 | 5.3 | 2.3 | 1.6 | 58 | 1.0 |
| ESE | 101.26 | 123.75 | 25.3 | 10.8 | 2.4 | 1.5 | 201 | 3.4 |
| SE | 123.76 | 146.25 | 34.1 | 14.5 | 2.0 | 1.0 | 362 | 6.0 |
| SSE | 146.26 | 168.75 | 27.2 | 10.7 | 1.9 | 0.9 | 236 | 3.9 |
| S | 168.76 | 191.25 | 25.8 | 9.6 | 1.0 | 0.6 | 45 | 0.8 |
| SSW | 191.26 | 213.75 | 25.2 | 9.7 | 0.9 | 0.5 | 31 | 0.5 |
| SW | 213.76 | 236.25 | 31.4 | 7.5 | 1.7 | 0.9 | 20 | 0.3 |
| WSW | 236.26 | 258.75 | 36.1 | 11.1 | 1.3 | 0.6 | 26 | 0.4 |
| W | 258.76 | 281.25 | 31.4 | 12.7 | 2.3 | 1.2 | 106 | 1.8 |
| WNW | 281.26 | 303.75 | 24.5 | 8.5 | 3.9 | 1.5 | 1522 | 25.4 |
| NW | 303.76 | 326.25 | 33.9 | 10.5 | 5.0 | 1.9 | 5716 | 95.3 |
| NNW | 326.26 | 248.75 | 30.2 | 14.1 | 4.1 | 1.9 | 835 | 13.9 |

**Table S2**: Distribution statistics for measurement averaging-intervals for the treatment arrays only.

| Array | Type | Skewness | Kurtosis | Q_1_ | Q_5_ | Q_25_ | Median | Q_75_ | Q_95_ | Q_99_ |
| --- | --- | --- | --- | --- | --- | --- | --- | --- | --- | --- |
| 1(f) | Grab | -2.04 | 11.6 | 253 | 100 | 136 | 147 | 157 | 175 | 193 |
| 1(f) | 1-min | -2.55 | 14.5 | 252 | 97 | 135 | 144 | 152 | 165 | 182 |
| 1(f) | 5-min | -2.67 | 15.0 | 251 | 98 | 135 | 144 | 151 | 164 | 180 |
| 4(f) | Grab | 0.62 | 38.7 | 533 | 102 | 129 | 140 | 153 | 171 | 190 |
| 4(f) | 1-min | 0.95 | 64.7 | 534 | 109 | 132 | 141 | 149 | 162 | 175 |
| 4(f) | 5-min | 0.90 | 65.0 | 538 | 107 | 132 | 142 | 149 | 161 | 175 |
| 6(f) | Grab | -0.66 | 19.2 | 403 | 108 | 132 | 144 | 155 | 173 | 191 |
| 6(f) | 1-min | -1.22 | 28.9 | 406 | 115 | 136 | 145 | 153 | 165 | 178 |
| 6(f) | 5-min | -1.22 | 31.3 | 454 | 115 | 136 | 145 | 152 | 165 | 178 |
| Mean | Grab | -0.69 | 22.9 | 533 | 103 | 132 | 144 | 155 | 173 | 190 |
| Mean | 1-min | -1.03 | 34.4 | 534 | 107 | 134 | 143 | 151 | 164 | 179 |
| Mean | 5 min | -1.08 | 35.5 | 538 | 106 | 134 | 144 | 151 | 163 | 178 |

**Table S3**. Summary statistics for the multiport atmospheric sampling system for the three treatment arrays 1(f), 4(f) and 6(f), over the 2017 operating season. X coordinates denotes horizontal positions moving from left to right (west to east) and y coordinates denotes horizontal positions moving up and down (north to south) in 6 m increments away from the array edge (central tower at 15 m). Z coordinate denotes the height component moving up through the three designated atmospheric layers.

| Array | x (m) | y (m) | z (m) | Average (µmol mol^-1^) | min (µmol mol^-1^) | max (µmol mol^-1^) | stdev (µmol mol^-1^) | Average per diff (%) | min per diff (%) | max per diff (%) | % Enrichment |
| --- | --- | --- | --- | --- | --- | --- | --- | --- | --- | --- | --- |
| 1(f) | 3 | 15 | 24 | 219 | 23 | 480 | 81 | 13 | -24 | 62 | 146 |
| 1(f) | 9 | 15 | 24 | 123 | 17 | 402 | 60 | -5 | -25 | 47 | 82 |
| 1(f) | 15 | 3 | 24 | 160 | 32 | 379 | 42 | 2 | -22 | 43 | 107 |
| 1(f) | 15 | 9 | 24 | 139 | 17 | 265 | 43 | -2 | -25 | 20 | 92 |
| 1(f) | 15 | 15 | 24 | 148 | 24 | 246 | 38 | 0 | -23 | 17 | 99 |
| 1(f) | 15 | 21 | 24 | 129 | 18 | 246 | 42 | -4 | -25 | 18 | 86 |
| 1(f) | 15 | 27 | 24 | 159 | 20 | 277 | 44 | 2 | -24 | 24 | 106 |
| 1(f) | 21 | 15 | 24 | 123 | 38 | 495 | 55 | -5 | -21 | 62 | 82 |
| 1(f) | 27 | 15 | 24 | 134 | 24 | 302 | 44 | -3 | -23 | 27 | 90 |
| 1(f) | 3 | 15 | 10 | 89 | 19 | 180 | 28 | -11 | -24 | 6 | 60 |
| 1(f) | 9 | 15 | 10 | 106 | 19 | 193 | 33 | -8 | -24 | 8 | 71 |
| 1(f) | 15 | 3 | 10 | 86 | 15 | 179 | 26 | -12 | -25 | 5 | 57 |
| 1(f) | 15 | 9 | 10 | 93 | 18 | 177 | 29 | -11 | -25 | 5 | 62 |
| 1(f) | 15 | 15 | 10 | 91 | 28 | 161 | 26 | -11 | -23 | 2 | 61 |
| 1(f) | 15 | 21 | 10 | 93 | 17 | 188 | 29 | -11 | -25 | 7 | 62 |
| 1(f) | 15 | 27 | 10 | 91 | 25 | 191 | 31 | -11 | -23 | 8 | 60 |
| 1(f) | 21 | 15 | 10 | 85 | 25 | 158 | 27 | -12 | -23 | 2 | 57 |
| 1(f) | 27 | 15 | 10 | 119 | 39 | 306 | 47 | -6 | -21 | 29 | 79 |
| 1(f) | 3 | 15 | 2 | 180 | 30 | 465 | 54 | 6 | -22 | 60 | 120 |
| 1(f) | 9 | 15 | 2 | 184 | 18 | 330 | 55 | 6 | -24 | 34 | 122 |
| 1(f) | 15 | 3 | 2 | 175 | 34 | 331 | 48 | 5 | -22 | 34 | 117 |
| 1(f) | 15 | 9 | 2 | 156 | 28 | 321 | 38 | 1 | -23 | 32 | 104 |
| 1(f) | 15 | 15 | 2 | 149 | 38 | 346 | 38 | 0 | -21 | 37 | 99 |
| 1(f) | 15 | 21 | 2 | 151 | 30 | 334 | 40 | 0 | -23 | 35 | 101 |
| 1(f) | 15 | 27 | 2 | 157 | 34 | 293 | 43 | 1 | -22 | 27 | 105 |
| 1(f) | 21 | 15 | 2 | 138 | 28 | 283 | 38 | -2 | -23 | 25 | 92 |
| 1(f) | 27 | 15 | 2 | 152 | 26 | 354 | 54 | 0 | -23 | 37 | 102 |
| 4(f) | 3 | 15 | 24 | 122 | 35 | 319 | 45 | -5 | -21 | 31 | 81 |
| 4(f) | 9 | 15 | 24 | 108 | 45 | 204 | 25 | -8 | -19 | 10 | 72 |
| 4(f) | 15 | 3 | 24 | 156 | 75 | 294 | 41 | 1 | -14 | 27 | 104 |
| 4(f) | 15 | 9 | 24 | 187 | 79 | 359 | 64 | 7 | -14 | 37 | 125 |
| 4(f) | 15 | 15 | 24 | 138 | 83 | 227 | 23 | -2 | -13 | 14 | 92 |
| 4(f) | 15 | 21 | 24 | 135 | 56 | 286 | 40 | -3 | -17 | 25 | 90 |
| 4(f) | 15 | 27 | 24 | 100 | 58 | 198 | 24 | -9 | -17 | 9 | 67 |
| 4(f) | 21 | 15 | 24 | 134 | 76 | 268 | 37 | -3 | -14 | 21 | 89 |
| 4(f) | 27 | 15 | 24 | 109 | 21 | 461 | 85 | -8 | -24 | 55 | 73 |
| 4(f) | 3 | 15 | 10 | 138 | 42 | 480 | 89 | -2 | -20 | 61 | 92 |
| 4(f) | 9 | 15 | 10 | 77 | 22 | 147 | 21 | -13 | -24 | -1 | 51 |
| 4(f) | 15 | 3 | 10 | 93 | 29 | 203 | 32 | -11 | -22 | 10 | 62 |
| 4(f) | 15 | 9 | 10 | 71 | -7 | 149 | 31 | -14 | -29 | 0 | 48 |
| 4(f) | 15 | 15 | 10 | 88 | 50 | 172 | 23 | -11 | -19 | 4 | 58 |
| 4(f) | 15 | 21 | 10 | 75 | 32 | 144 | 20 | -14 | -22 | -1 | 50 |
| 4(f) | 15 | 27 | 10 | 79 | 12 | 245 | 30 | -13 | -25 | 18 | 52 |
| 4(f) | 21 | 15 | 10 | 72 | 13 | 148 | 24 | -14 | -26 | 0 | 48 |
| 4(f) | 27 | 15 | 10 | 70 | -17 | 195 | 40 | -15 | -31 | 8 | 47 |
| 4(f) | 3 | 15 | 2 | 111 | 28 | 291 | 41 | -7 | -22 | 26 | 74 |
| 4(f) | 9 | 15 | 2 | 76 | 30 | 150 | 22 | -14 | -23 | 0 | 50 |
| 4(f) | 15 | 3 | 2 | 125 | 57 | 266 | 43 | -5 | -17 | 21 | 83 |
| 4(f) | 15 | 9 | 2 | 110 | 42 | 238 | 39 | -7 | -20 | 16 | 73 |
| 4(f) | 15 | 15 | 2 | 94 | 37 | 222 | 31 | -10 | -21 | 13 | 63 |
| 4(f) | 15 | 21 | 2 | 110 | 36 | 231 | 38 | -7 | -21 | 14 | 73 |
| 4(f) | 15 | 27 | 2 | 104 | 24 | 232 | 38 | -8 | -22 | 15 | 70 |
| 4(f) | 21 | 15 | 2 | 93 | 33 | 254 | 43 | -11 | -22 | 18 | 62 |
| 4(f) | 27 | 15 | 2 | 100 | 33 | 355 | 60 | -9 | -22 | 37 | 67 |
| 6(f) | 3 | 15 | 24 | 174 | 36 | 650 | 111 | 4 | -21 | 92 | 116 |
| 6(f) | 9 | 15 | 24 | 178 | 73 | 781 | 95 | 5 | -14 | 116 | 118 |
| 6(f) | 15 | 3 | 24 | 172 | 32 | 640 | 98 | 4 | -22 | 90 | 115 |
| 6(f) | 15 | 9 | 24 | 170 | 71 | 718 | 83 | 4 | -15 | 104 | 113 |
| 6(f) | 15 | 15 | 24 | 151 | 95 | 693 | 72 | 0 | -10 | 100 | 101 |
| 6(f) | 15 | 21 | 24 | 140 | 68 | 652 | 76 | -2 | -15 | 92 | 93 |
| 6(f) | 15 | 27 | 24 | 86 | 11 | 488 | 67 | -12 | -25 | 62 | 58 |
| 6(f) | 21 | 15 | 24 | 152 | 62 | 589 | 75 | 0 | -16 | 81 | 101 |
| 6(f) | 27 | 15 | 24 | 138 | 39 | 513 | 80 | -2 | -20 | 67 | 92 |
| 6(f) | 3 | 15 | 10 | 95 | 16 | 525 | 63 | -10 | -24 | 69 | 63 |
| 6(f) | 9 | 15 | 10 | 111 | 52 | 565 | 64 | -7 | -18 | 76 | 74 |
| 6(f) | 15 | 3 | 10 | 138 | 39 | 574 | 73 | -2 | -20 | 78 | 92 |
| 6(f) | 15 | 9 | 10 | 133 | 43 | 557 | 67 | -3 | -19 | 75 | 88 |
| 6(f) | 15 | 15 | 10 | 106 | 31 | 532 | 60 | -8 | -22 | 70 | 71 |
| 6(f) | 15 | 21 | 10 | 90 | 18 | 492 | 58 | -11 | -24 | 63 | 60 |
| 6(f) | 15 | 27 | 10 | 73 | 5 | 458 | 57 | -14 | -26 | 56 | 49 |
| 6(f) | 21 | 15 | 10 | 100 | 33 | 538 | 59 | -9 | -21 | 71 | 67 |
| 6(f) | 27 | 15 | 10 | 99 | 22 | 498 | 63 | -9 | -23 | 64 | 66 |
| 6(f) | 3 | 15 | 2 | 175 | 50 | 678 | 94 | 5 | -18 | 97 | 116 |
| 6(f) | 9 | 15 | 2 | 147 | 70 | 607 | 72 | -1 | -14 | 84 | 98 |
| 6(f) | 15 | 3 | 2 | 156 | 42 | 609 | 83 | 1 | -19 | 84 | 104 |
| 6(f) | 15 | 9 | 2 | 152 | 62 | 617 | 76 | 0 | -16 | 86 | 101 |
| 6(f) | 15 | 15 | 2 | 148 | 79 | 590 | 71 | 0 | -13 | 81 | 99 |
| 6(f) | 15 | 21 | 2 | 147 | 52 | 578 | 71 | -1 | -18 | 79 | 98 |
| 6(f) | 15 | 27 | 2 | 136 | 40 | 524 | 71 | -3 | -21 | 69 | 91 |
| 6(f) | 21 | 15 | 2 | 143 | 59 | 582 | 68 | -1 | -16 | 79 | 95 |
| 6(f) | 27 | 15 | 2 | 147 | 63 | 573 | 67 | 0 | -16 | 78 | 98 |
